# Association Between Diabetes Prevalence and Mortality from Gastrointestinal Cancers in Türkiye: A Longitudinal Ecological Study

**DOI:** 10.3390/jcm15156037

**Published:** 2026-08-03

**Authors:** Muammer Bilici, Güray Ceylan, Kadir Yılmaz

**Affiliations:** 1 Internal Medicine, Bülent Ecevit University, 67100 Zonguldak, Türkiye; drmbilici@hotmail.com (M.B.); guray.ceylan@beun.edu.tr (G.C.); 2 Department of Statistics, Istanbul Commerce University, 34445 İstanbul, Türkiye

**Keywords:** diabetes, digestive system, cancer, mortality

## Abstract

**Background/Objectives**: This study aimed to analyze the effect of diabetes prevalence on mortality levels from gastrointestinal cancers. **Methods**: The study utilized mortality data for Türkiye from the World Health Organization (WHO) Mortality Database, covering deaths from stomach, colon and rectum, liver, and pancreas cancers, as well as diabetes prevalence data, between 2009 and 2023. Health expenditure and life expectancy data from the World Bank were used as control variables. **Results**: Diabetes prevalence was significantly correlated with colon and rectum cancer mortality (r = 0.929; *p* < 0.01), pancreas cancer mortality (r = 0.978; *p* < 0.01), life expectancy (r = 0.941; *p* < 0.01), and health expenditure (r = −0.635; *p* < 0.01). Correlations of diabetes prevalence with esophagus, stomach, and liver cancer mortality were statistically insignificant (*p* > 0.05). According to generalized linear model analysis results, there was an effect of diabetes prevalence on colon and rectum cancer mortality (B = 383.108; *p* < 0.01) and pancreas cancer mortality (B = 330.415; *p* < 0.01). Effects of diabetes prevalence on esophagus, stomach, and liver cancer mortality were statistically insignificant (*p* > 0.05). **Conclusions**: Diabetes prevalence was significantly associated with mortality levels for colon and rectal cancer and pancreatic cancer; however, it did not have a significant effect on mortality levels for esophageal, gastric, and liver cancers. Although there is a close relationship between diabetes and the digestive system, a similar relationship between cancer mortality levels of digestive system organs and diabetes is not valid for every organ. We believe that the effect of diabetes on gastrointestinal cancers has a tissue- or organ-specific mechanism of action rather than a digestive mechanism.

## 1. Introduction

Diabetes, a metabolic and autoimmune disease characterized by hyperglycemia, is one of the major health problems today and has two main types: type 1 and type 2 diabetes [1,2]. Especially in recent years, type 2 diabetes has an increasing prevalence in different geographies and regions [3]. Obesity, genetic factors, diet, and other demographic characteristics are reported as risk factors for diabetes [4,5,6]. On the other hand, diabetes itself is a risk factor for many cardiovascular and systemic chronic diseases [7,8,9]. Although the direct relationship between diabetes and mortality or diabetes-related death rates is low, diabetes constitutes an important risk factor for the prognosis and mortality of many diseases [10,11]. Gastrointestinal cancers are also among the diseases where a relationship between diabetes and mortality has been reported and where the relationship between diabetes and nutrition is highlighted.

Gastrointestinal cancers, which include cancers of the mouth, throat, esophagus, stomach, small intestine, and colorectum, still have significant mortality rates today, even though early diagnosis and treatment options have improved [12]. Although each of these cancer types has a different pathophysiology, they can be studied in a group compared to other different organ cancer types because they are included in the same system [13,14,15]. In their study of the six most important gastrointestinal cancers, Zhou et al. [16] reported that colorectal cancer had the highest mortality and morbidity among them and that gastrointestinal cancers in general constitute a significant burden on the health system. The relationship between diabetes and digestive system health and the relationship between gastrointestinal cancer types can be used as an indicator to predict cancer levels in these organs, with diabetes being a significant risk factor. However, this requires research at large sample and population levels. Although there are studies in the literature that establish a link between gastrointestinal cancers and diabetes, there is a lack of sufficient research specifically on the relationship between gastrointestinal cancer and diabetes. Therefore, this study aimed to analyze the effect of diabetes prevalence on gastrointestinal cancer mortality levels. Although cancers of the mouth, throat, esophagus, stomach, small intestine, and colorectum are also considered digestive system cancers, WHO data specifically track esophagus, stomach, colon/rectum, liver, and pancreas cancers. Therefore, this study analyzed data from these specific cancer types.

## 2. Materials and Methods

### 2.1. Research Model

The study was designed as a retrospective longitudinal ecological study based on aggregated annual data. First, gastrointestinal cancer mortality rates, diabetes, life expectancy, and healthcare expenditures were defined. Then, the effects of diabetes and the control variables (healthcare expenditures and life expectancy) on the mortality level of each gastrointestinal cancer were analyzed.

### 2.2. Research Data

In this study, mortality data for stomach, colon and rectum, liver, and pancreatic cancer, as well as diabetes prevalence data, were obtained from the World Health Organization (WHO) Mortality Database for Türkiye between 2009 and 2023. Health expenditure and life expectancy data from the World Bank were used as control variables. Because all cases from all years provided by the WHO were included in the analysis, a complete enumeration sample was used, and power analysis was not performed. Because the data were prevalence data, exclusion and inclusion criteria were not applicable. No values were interpolated or extrapolated. The distribution of the data in the study was as follows:

WHO data:

- Diabetes, prevalence (%) (https://www.who.int/data/gho/data/indicators/indicator-details/GHO/prevalence-of-diabetes-age-standardized, accessed on 23 June 2026).
- Esophagus (ICD 10-C15), stomach (ICD 10-C16), colon/rectum (ICD 10-C18, C19, C20), liver (ICD 10-C22), and pancreas (ICD 10-C25) cancer deaths (https://platform.who.int/mortality/themes/theme-details/topics/topic-details/MDB/malignant-neoplasms, accessed on 23 June 2026).

World Bank data:

- Life expectancy at birth, total (years) (SP.DYN.LE00.IN).
- Current health expenditure (% of GDP) (SH.XPD.CHEX.GD.ZS).
- Population growth (annual %) (SP.RUR.TOTL.ZG).

Research parameters according to years were given in the Table 1. 

In order to demonstrate the temporal effect, we added population size variation, as suggested by both Reviewer 1 and Reviewer 2. WHO and World Bank data on variance inflation factors or condition indices do not include deflators; however, for this effect, we always used the variables as percentages. For example, we used health expenditure as a percentage of GDP and population growth as a percentage.

### 2.3. Ethical Approval and Informed Patient Consent

Because the research data were made public by the WHO and the World Bank without requiring any usage permission, and because the data were in the form of prevalence figures and did not contain personal information, ethics committee approval and informed patient consent were not obtained. The writing process was carried out in accordance with the Declaration of Helsinki and Good Clinical Practice guidelines.

### 2.4. Statistical Methods

Measurement parameters were defined by the mean, standard deviation, median, and range of variation. The Kolmogorov–Smirnov test was performed for normality distribution. Due to the proportional nature of the data and the application of a nonparametric logit model, unit root and autocorrelation tests were not performed. Spearman’s rho nonparametric correlation analysis was performed for relational analysis. For effect analysis, generalized linear model (logit) analysis was performed due to linearization deviations [17,18]. All analyses were performed in SPSS 25.0 for Windows at a significance level of 0.05 and a 95% confidence interval. In this study, alternative time-series approaches, including Unit Root Tests (ADF and PP) and ARIMA modeling, were methodologically assessed for the analysis of prevalence data. However, because prevalence rates are inherently nonlinear and strictly bounded within the (0, 1) interval, standard linear ARIMA frameworks and ADF/PP unit root assumptions can mathematically generate invalid out-of-bound predictions, such as negative or above 100% values. To bypass these time-series constraints and respect the true distributional properties of the data, a generalized linear model (GLM) framework was employed. By adjusting for potential autocorrelation within the model’s design, this GLM approach provides highly robust and reliable parameter estimates, eliminating the need for an ARIMA model. Dependent variables in each model were gastrointestinal cancer mortality, whereas the independent variable was diabetes prevalence and control variables were health expenditure, life expectancy, and population growth. Annual numbers of gastrointestinal cancers were modeled using an independent GLM model. A Poisson distribution with a log-linked function was assumed for the count response variable. To control for the possible presence of overdistribution, the dispersion parameter was formally assessed using the ratio of the Pearson chi-square statistic to the residual degrees of freedom. To account for changing population sizes over the years and to convert the numbers to standardized proportions, the natural logarithm of the annual mid-year population from the World Bank database was included as a population adjustment. Potential serial autocorrelation arising from the longitudinal, time-series nature of the annual data was addressed using Newey–West standard errors to ensure robust statistical inference. The analysis was based on a total of 15 years of observation, and model fit was assessed using residual deviation.

## 3. Results

Diabetes prevalence ranged from 9.50% to 16.20%, with a 12.55 ± 2.18% mean value. Mean cancer mortality was 768.80 ± 73.42 for esophagus cancer, 6178.00 ± 574.36 for stomach cancer, 6715.33 ± 969.06 for colon and rectum cancer, 2727.93 ± 346.30 for liver cancer, and 4453.73 ± 763.53 for pancreas cancer. Life expectancy mean was 75.16 ± 1.81 years, and health expenditure mean was 4.65 ± 0.44% of GDP (Table 2).

**Figure 1:**
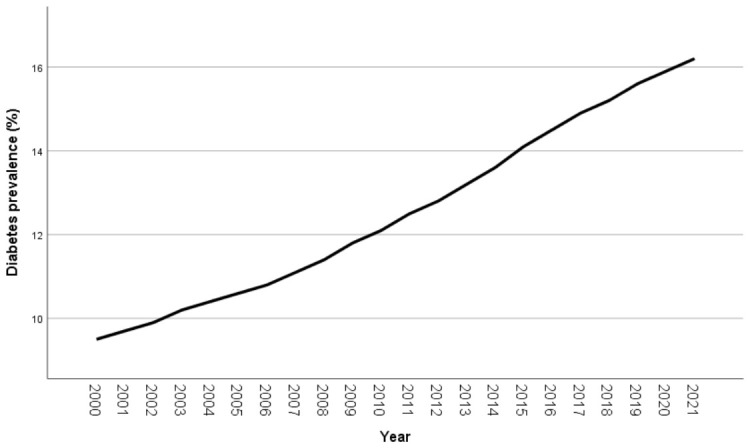
Diabetes prevalence change according to years.

From 2000 to 2021, diabetes prevalence in Türkiye had an increasing trend with a stable slope (Figure 1).

**Figure 2:**
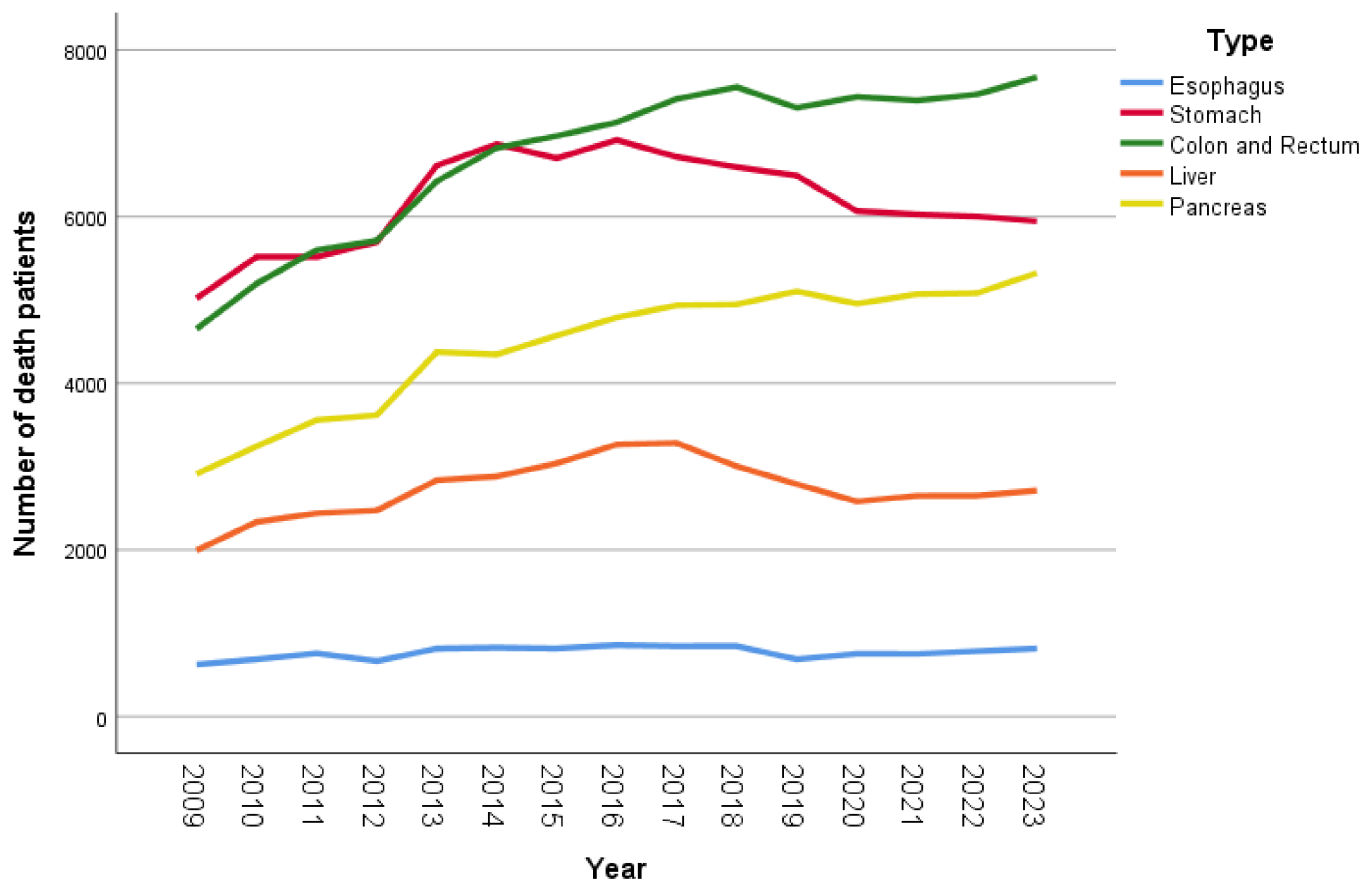
Changes in gastrointestinal cancer mortality according to year and type.

Although stomach cancer mortality had the highest level in 2009, colon and rectum cancer mortality was the highest in 2023. All gastrointestinal cancer types increased during the 2009 to 2023 time period (Figure 2).

Diabetes prevalence was significantly correlated with colon and rectum cancer mortality (r = 0.929; *p* < 0.01), pancreas cancer mortality (r = 0.978; *p* < 0.01), life expectancy (r = 0.941; *p* < 0.01), and health expenditure (r = −0.635; *p* < 0.01). Correlations of diabetes prevalence with esophagus, stomach, and liver cancer mortality were statistically insignificant (*p* > 0.05) (Table 3).

According to generalized linear model analysis results, there were effect of diabetes prevalence on colon and rectum cancer mortality (B = 383.108; *p* < 0.01) and pancreas cancer mortality (B = 330.415; *p* < 0.01). Effect of diabetes prevalence on esophagus, stomach, and liver cancer mortality were statistically insignificant (*p* > 0.05) (Table 4).

## 4. Discussion

This study aimed to analyze the effect of diabetes prevalence on mortality levels of gastrointestinal cancers and analyzed mortality levels of gastrointestinal cancer types in Türkiye between 2009 and 2023. According to the results, diabetes prevalence was significantly associated with mortality levels of colon and rectal cancer and pancreatic cancer; however, it did not have a significant effect on mortality levels of esophageal, gastric, or liver cancers.

Diabetes, a metabolic and autoimmune disease with increasing prevalence and incidence, particularly for type 2, is closely related to the digestive system [1,2,3,4,5,6]. The relationship between diabetes and the digestive system is bidirectional, with digestive system diseases triggering diabetes and diabetes affecting diseases of the digestive system organs [8,9,10,11,12]. Therefore, the prevalence of diabetes may also have an impact on mortality levels in gastrointestinal cancers. However, it can be stated that studies in the literature on this subject are insufficient either conceptually or in terms of content and patient numbers. For this reason, it would be beneficial to examine the issue at the population level and from a broader perspective in a multidisciplinary manner.

In one of the limited studies on gastrointestinal cancers and diabetes, Chai et al. [19] conducted a meta-analysis of 84 trials and 101,595 patients and reported that incretin-based treatments increased the risk of gastrointestinal cancer in type 2 diabetes patients. Hashimoto et al. [20] examined the effects of hypertension and diabetes on gastrointestinal cancer in elderly individuals and reported that hypertension and diabetes may be risk factors for multiple organs in gastrointestinal cancers. Rathmann and Kostev [21] examined the effects of type 2 diabetes on breast, prostate, and gastrointestinal cancers in patients with the disease and reported that the drugs used for treatment may be associated with gastrointestinal cancer. Valent [22] reported that gastrointestinal cancers increased with diabetes in his study on an Italian population. Matthews et al. [23] reported that diabetes has an effect on mortality in patients with metabolic syndrome and gastrointestinal cancer. Hillon et al. [24] reported that obesity and type 2 diabetes are associated with gastrointestinal cancer. In their study, Wang et al. [25] reported that diabetes and lifestyle had a significant effect on gastrointestinal cancers.

In our study, diabetes prevalence was significantly associated with mortality levels for colon and rectal cancer and pancreatic cancer; however, it did not have a significant effect on mortality levels for esophageal, gastric, and liver cancers. These population-level results indicate that diabetes does not affect all gastrointestinal organs similarly but rather has a more effective mechanism, particularly in colon, rectal, and pancreatic cancer. Further research is needed to understand the specific mechanisms through which this effect varies.

Table 3 shows a statistically significant negative association between health expenditure and mortality across nearly all cancer types (e.g., B = −931.259 for stomach cancer, B = −831.651 for colorectal cancer). This demonstrates the effectiveness of health expenditures in combating cancer mortality levels. Furthermore, further studies are needed to analyze what changes in cancer treatment cost over time, the increase in available resources, and the development of screening and early detection methods.

All variables used in the research are national-level aggregates of prevalence and mortality counts, not individual-level data. Associations observed at this level cannot be assumed to hold at the individual patient level. Therefore, research results should be interpreted at the population level, not directly at the individual level.

### 4.1. Limitations of the Study

The most significant limitation of the study is that, due to its population-based nature, the data are presented as prevalence data. Therefore, many variables that influence gastrointestinal cancer and mortality levels in clinical trials, such as gender, economic status, age, comorbidities, history of medication use, and nutritional level, are not applicable to population-level data. Our study includes time series with varying numbers of patients each year, but always including all patients and mortality levels. While this is a limitation compared to clinical trials, it constitutes a fundamental resource for further studies.

Another significant limitation of the study is the lack of sufficient population-level and multivariate analyses in the literature regarding the relationship between gastrointestinal cancer mortality levels and diabetes. This results in limitations both in terms of time series analysis and comparison of results. While the literature mentions a correlation between gastrointestinal cancer mortality levels and diabetes, these are generally studies that focus on either a specific gastrointestinal cancer or consider diabetes as comorbidity within mortality groups. In our study, however, diabetes was analyzed as a risk factor in both univariate and multivariate analyses.

The ecological fallacy is a distinct and fundamental limitation of the study design, and research results should not be correlated with individual mortality cases. In clinical practice, findings should be considered in terms of population trends, not on a patient-by-patient basis.

### 4.2. Contribution of the Study to the Literature and Clinical Practice

The most important contribution of the study to the literature is that it addresses the different results presented by various studies in clinical research in a holistic manner and at the population level, with highly evidence-based and generalizable evidence. In this respect, our study describes the general framework by both including all patients and examining the subject at the population level and by considering all gastrointestinal cancers. In this respect, our study both fills a gap in the literature and serves as a resource for further studies.

The contribution of this research to clinical practice is that it reveals the risk level of diabetes, especially in patients with colon and rectal cancer and pancreatic cancer, demonstrating the need for closer monitoring in these patients. The significantly higher mortality rate in these patients points to an organ–diabetes related mechanism. Therefore, it is beneficial to examine all disease parameters that may be related to this mechanism more closely than other parameters during follow-up in these patients.

## 5. Conclusions

According to our study, diabetes prevalence was significantly associated with mortality levels for colon and rectal cancer and pancreatic cancer; however, it did not have a significant effect on mortality levels for esophageal, gastric, or liver cancers. Although there is a close relationship between diabetes and the digestive system, a similar relationship between cancer mortality levels in digestive system organs and diabetes is not valid for every organ. At the population level, increasing diabetes prevalence was associated with higher colorectal and pancreatic cancer mortality in Türkiye, whereas no statistically significant associations were observed for esophageal, gastric, or liver cancer mortality. These ecological findings are hypothesis-generating and should be confirmed in individual-level longitudinal studies that account for demographic, metabolic, lifestyle, and cancer-related confounders.

## Figures and Tables

**Table 1:** Research parameters according to years.

Year	DM	Health Expenditure	Esophagus	Stomach	Colon and Rectum	Liver	Pancreas	Life Expectancy	Population Growth
2009	11.80	5.49	623	5018	4652	1995	2910	74,679	1.38
2010	12.10	5.02	687	5516	5195	2335	3243	74,964	1.52
2011	12.50	4.65	758	5516	5598	2439	3557	74,823	1.47
2012	12.80	4.44	666	5694	5710	2472	3616	75,552	1.27
2013	13.20	4.37	816	6611	6422	2835	4373	76,171	1.28
2014	13.60	4.33	826	6868	6823	2880	4345	76,452	1.35
2015	14.10	4.12	817	6700	6967	3038	4571	76,539	1.33
2016	14.50	4.28	857	6917	7130	3265	4788	76,595	1.35
2017	14.90	4.18	844	6715	7411	3281	4935	77,037	1.30
2018	15.20	4.12	845	6591	7552	3002	4944	77,454	1.35
2019	15.60	4.36	687	6491	7304	2788	5103	77,737	1.43
2020	15.90	4.62	753	6065	7437	2580	4954	76,525	0.97
2021	16.20	4.56	752	6025	7392	2648	5067	75,722	0.91
2022		3.70	784	6000	7465	2650	5079	77,591	0.98
2023		4.28	817	5943	7672	2711	5321	77,156	0.41

**Table 2:** Descriptive statistics for diabetes prevalence, gastrointestinal cancer mortality, life expectancy, and health expenditure.

	Mean	Standard Deviation	Median	Minimum	Maximum
Diabetes prevalence, %	12.55	2.18	12.30	9.50	16.20
Esophagus cancer mortality, patients	768.80	73.42	784.00	623.00	857.00
Stomach cancer mortality, patients	6178.00	574.36	6065.00	5018.00	6917.00
Colon and rectum cancer mortality, patients	6715.33	969.06	7130.00	4652.00	7672.00
Liver cancer mortality, patients	2727.93	346.30	2711.00	1995.00	3281.00
Pancreas cancer mortality, patients	4453.73	763.53	4788.00	2910.00	5321.00
Life expectancy, years	75.16	1.81	75.26	71.93	77.74
Health expenditure, % of GDP	4.65	0.44	4.61	3.70	5.49
Population growth, annual %	1.12	0.36	1.26	0.16	1.52

**Table 3:** Spearman’s rho correlation between diabetes prevalence, gastrointestinal cancer mortality, life expectancy, and health expenditure.

Diabetes Prevalence, %	r	*p*
Esophagus cancer mortality, patients	0.338	0.258
Stomach cancer mortality, patients	0.421	0.152
Colon and rectum cancer mortality, patients	0.929 **	0.000
Liver cancer mortality, patients	0.505	0.078
Pancreas cancer mortality, patients	0.978 **	0.000
Life expectancy, years	0.941 **	0.000
Health expenditure, % of GDP	−0.635 **	0.001
Population growth, annual %	0.266	0.232

** *p* < 0.01.

**Table 4:** Generalized linear model (logit) for the effect of diabetes prevalence on gastrointestinal cancer mortality.

Parameters	B *	Std. Error	95% Wald Confidence Interval		Hypothesis Test		
			Lower	Upper	Wald X^2^	df	*p*
***Esophagus***							
Diabetes prevalence, %	0.321	26.1557	−50.944	51.585	0.000	1	0.990
Life expectancy, years	−10.678	36.6807	−82.571	61.214	0.085	1	0.771
Health expenditure, % of GDP	−175.302	55.6900	−284.452	−66.151	9.909	1	0.002
Population growth, annual %	−4.476	148.3313	−295.200	286.248	0.001	1	0.976
***Stomach***							
Diabetes prevalence, %	−118.970	135.6466	−384.833	146.892	0.769	1	0.380
Life expectancy, years	363.657	190.2304	−9.188	736.501	3.654	1	0.056
Health expenditure, % of GDP	−907.531	288.8145	−1473.597	−341.465	9.874	1	0.002
Population growth, annual %	−476.893	769.2630	−1984.621	1030.835	0.384	1	0.535
***Colon and rectum***							
Diabetes prevalence, %	383.108	78.9045	228.458	537.758	23.574	1	0.000
Life expectancy, years	171.472	110.6554	−45.408	388.353	2.401	1	0.121
Health expenditure, % of GDP	−815.374	168.0009	−1144.649	−486.098	23.555	1	0.000
Population growth, annual %	−327.136	447.4735	−1204.168	549.896	0.534	1	0.465
***Liver***							
Diabetes prevalence, %	26.420	85.8687	−141.880	194.719	0.095	1	0.758
Life expectancy, years	54.892	120.4220	−181.130	290.915	0.208	1	0.649
Health expenditure, % of GDP	−703.637	182.8289	−1061.975	−345.299	14.812	1	0.000
Population growth, annual %	228.373	486.9682	−726.067	1182.813	0.220	1	0.639
***Pancreas***							
Diabetes prevalence, %	330.415	64.3052	204.379	456.451	26.401	1	0.000
Life expectancy, years	138.161	90.1815	−38.592	314.913	2.347	1	0.126
Health expenditure, % of GDP	−482.715	136.9167	−751.066	−214.363	12.430	1	0.000
Population growth, annual %	−154.862	364.6802	−869.622	559.898	0.180	1	0.671

* change in mortality count per 1 unit/1% change in diabetes prevalence.

## Data Availability

The data that support the findings of this study are publicly available from the World Health Organization and World Bank Country Reports on their official websites.

## Abbreviations

The following abbreviations are used in this manuscript:

GDP	Gross Domestic Product
WHO	World Health Organization
